# Ewing’s Sarcoma of the Adrenal Gland

**DOI:** 10.21699/ajcr.v7i3.406

**Published:** 2016-06-15

**Authors:** Dilip Kumar Pal, Vipin Chandra, Kumar Rajiv Ranjan, Debasis Chakrabortty, Manju Banerjee

**Affiliations:** 1 Department of Urology, Institute of Post Graduate Medical Education and Research, Kolkata; 2 Department of Pathology, Institute of Post Graduate Medical Education and Research, Kolkata; 3 Department of Surgery, Institute of Post Graduate Medical Education and Research, Kolkata

**Keywords:** Ewing's sarcoma, Primitive neuro-ectodermal tumor, Arenal tumor, Adrenal gland

## Abstract

Ewing’s sarcoma (ES) or primitive neuro-ectodermal tumor (PNET) typically occurs in long or flat bones, the chest wall, extra-skeletal soft tissue, and rarely in solid organs. Incidence of adrenal Ewing’s sarcoma is very rare. Here we report a case of Ewing’s sarcoma of the right adrenal gland in an 8-year-old girl who presented with an abdominal mass. The huge tumor was managed by preoperative neo-adjuvant chemotherapy followed by surgical resection. She died due to metastasis after five months of surgery.

## CASE REPORT

An 8-year-old girl presented with progressive pain and palpable lump in right flank for the last one month. Ultrasonography (USG) showed right retroperitoneal tumor, suprarenal in location. Contrast enhanced computed tomography (CECT) demonstrated 12.7 cm x 11.8 cm large variegated mass in right adrenal gland with retroperitoneal lymphadenopathy (Fig. 1). A 24-hour urinary catecholamines, plasma metanephrine, aldosterone, and plasma renin levels were within normal range. CT-guided core biopsy revealed malignant small round cell tumor. Bone-marrow aspiration was unremarkable. Tumor was large and appeared unresectable thus neoadjuvant chemotherapy was planned and given for seven cycles using vincristine, doxorubicin and cyclophosphamide alternating with ifosfamide/etoposide with granulocyte colony stimulating factor (GCSF). Tumor regressed to one-fourth of its original size and lymph nodes appeared insignificant size (Fig. 2). Patient also improved symptomatically. At operation the tumor was found densely adherent to surrounding tissues especially the upper pole of right kidney and inferior vena cava (Fig. 2). The tumor was ultimately resected. Grossly the specimen was 8 cm x 6 cm with tan-yellow cut surface and areas of hemorrhage and necrosis. Histopathology showed adrenal medulla and cortex completely replaced by a small round blue cell tumor (Fig. 3A). Immunohistochemical (IHC) studies showed that tumor cells were positive for CD99 (membranous), synaptophysin and chromogranin (Fig. 3B). Postoperatively the patient was lost to follow up and admitted after five months with metastasis in lungs and liver and died within few days.

**Figure:**
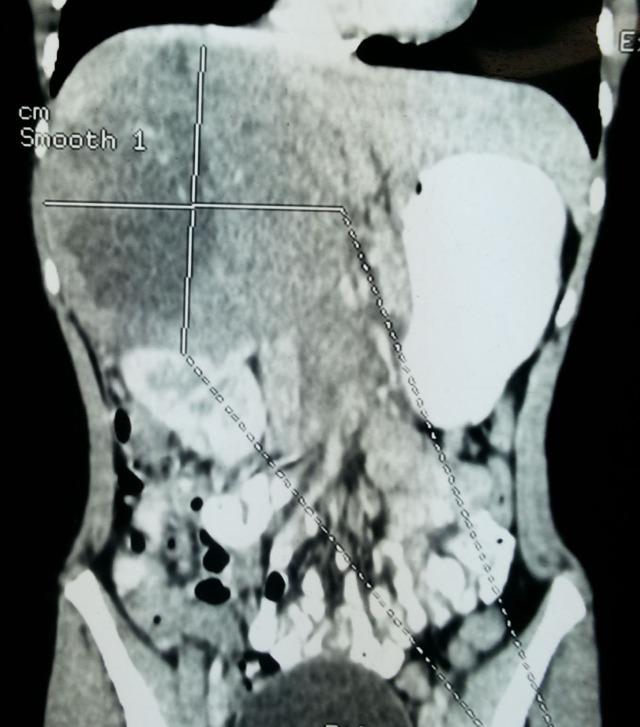
Figure 1:CECT showing huge adrenal tumor.

**Figure:**
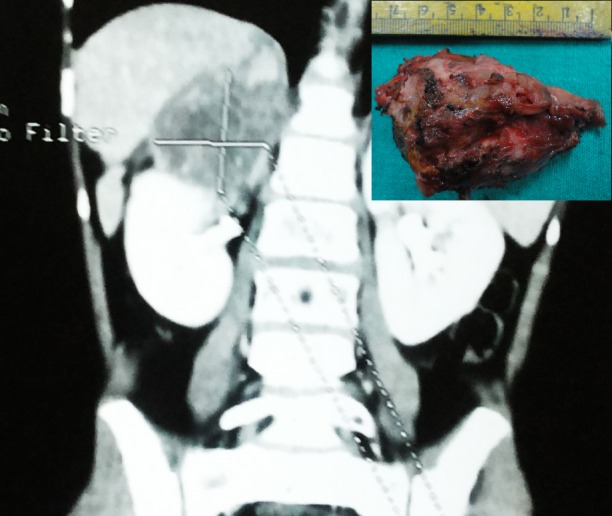
Figure 2:CECT of adrenal tumor after chemotherapy. Inset shows excised tumor.

**Figure:**
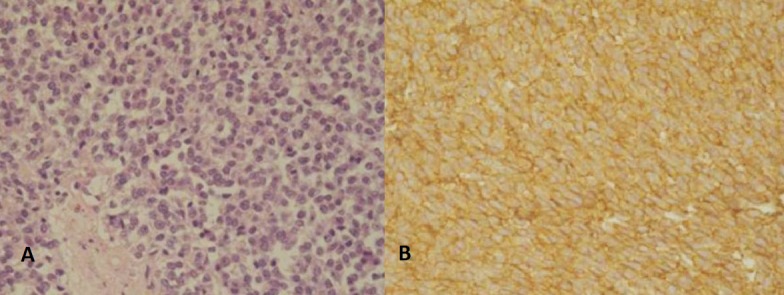
Figure 3:(A) Histopathology showing small rounded cells (H and Ex400); (B) IHC showing positive status with CD99.

## DISCUSSION

Ewing’s sarcoma/PNET of the adrenal gland is extremely rare.[1-3] Usually the patients present with flank pain and rapidly growing mass. USG should be done as an initial screening tool but CECT is essential to find out the relation with the surrounding structures. CECT imaging helps in distinguishing between benign and malignant adrenal tumors but exact nature.[4] Hormonal studies should be done to differentiate it from the functional adrenal tumors. It is difficult to make a definitive diagnosis of ES/PNET even on histopathology without IHC.[5] Our patient had very large tumor and considered non-resectable, so needle biopsy was done to find out the nature of the tumor. Final diagnosis was made after IHC. CD 99 is a highly sensitive marker for PNET/ES. Patients of ES/PNET show reciprocal translocation of t(11;22) (q24;q12) involving the EWSR1 gene on chromosome 22 and the FLI1 gene on chromosome 11 in more than 90% of cases but this is not routinely assessed.[2] As per available literature these tumors are very rapidly growing with aggressive nature. As this is a rare tumor, there is no definite guideline for management but surgical resection is considered to be the mainstay for local disease control.[5-7] This tumor is considered as radio and chemo sensitive so adjuvant chemotherapy and radiotherapy are advocated for better disease control but there is no consensus on standard chemo-radiation regimen.[8] We initially tried chemotherapy which worked well in our patient. The outcome seems good in case of complete excision but the prognosis was poor in our case. Proper postoperative surveillance with imaging modalities and adjuvant chemotherapy might alter the outcome in index case.

## Footnotes

**Source of Support:** Nil

**Conflict of Interest:** None declared

